# Predictive growth modeling of Yersinia enterocolitica in fresh kimchi cabbage brassica pekinensis as a function of storage temperature

**DOI:** 10.1016/j.heliyon.2023.e17978

**Published:** 2023-07-05

**Authors:** Sung-Hee Park, Ji Yoon Kim, Eun Hae Kim, Sung Gi Min, Shin Young Park

**Affiliations:** a PracticalTechnology Research Group, World Institute of Kimchi, Gwangju, 61755, Republic of Korea; b Department of Seafood Science and Technology, Institute of Marine Industry, Gyeongsang National University, Tongyeong, 53064, Republic of Korea

**Keywords:** Baranyi model, Kimchi cabbage, Predictive growth model, Storage temperatures, *Y. enterocolitica*

## Abstract

We developed a predictive growth model of *Yersinia enterocolitica* for fresh Kimchi cabbages as a function of storage temperature (5–20 °C). The Baranyi equation used for primary modeling at these storage temperatures was suitable as a model for obtaining lag time (LT) and specific growth rate (SGR) (R^2^ = 0.97–0.98). As the temperature increased, the growth of *Y. enterocolitica* tended to increase, with SGR values of 0.33, 0.40, 0.60 and 0.68 log colony-forming units/h at 8, 11, and 15 °C, and LT values of 5.63, 3.54, 2.23 and 1.09 h, respectively. The secondary model was determined by the non-linear regression analysis. The suitability of the modeling results for the SGR and LT value was verified by determining the mean square error (<0.01), bias factor (0.919–0.999), and accuracy factor (1.032–1.136). The predicted models can be used to predict the growth of *Y. enterocolitica* in Kimchi cabbage at various temperatures and as an effective tool for maintaining the safe level of *Y. enterocolitica* in the production, processing, and distribution of fresh agricultural products.

## 1 Introduction

Recently, predictive food microbiology has been actively studied as an approach for controlling and managing pathogenic microorganisms that cause food poisoning [1]. Predictive microbiology provides a mathematical model for the proliferation, growth, and death of food-poisoning bacteria during the exposure assessment phase of microbial risk assessment to quantitatively evaluate food hazards [2]. Predictive microbiology is related to internal factors such as water activity, pH and NaCl content and external factors such as the storage temperature, humidity, and nutrition that affect microbial growth throughout the production, distribution, consumption and storage processes [3]. In addition, these studies have attracted attention for preventing and controlling risk factors in advance by predicting bacterial growth using mathematical and statistical approaches.

Kimchi cabbage is a biennial plant (vegetable) belonging to the Brassicaceae family and is a major crop accounting for more than half of Korea's leafy vegetable production [4]. Kimchi cabbage is the main ingredient of kimchi, an important side dish in the Korean diet, and is used as an ingredient in various dishes such as steamed cabbage and cabbage pancake. The increase in single-person households, growth of the restaurant industry, increase in group meals, and change in food culture pursuing a simple diet have led to rapid increases in the demand for kimchi products and salted cabbage [5]. However, agricultural products grown in soil, such as Kimchi cabbage, are highly likely to be contaminated by soil-borne bacteria, and these microorganisms can survive in gaps and cracks and grow at high concentrations inside Kimchi cabbage. Particularly, fresh agricultural products, such as cabbage kimchi are consumed without a heating, increasing the risk of food poisoning caused by various pathogenic microorganisms.

*Yersinia enterocolitica* is a zoonotic pathogen that causes acute gastroenteritis and more serious diseases in humans [6]. Although the optimum temperature for the growth of *Y. enterocolitica* is 22–29 °C, the bacterium can survive at low temperatures below 10 °C, raising concerns regarding food safety [7]. As a food poisoning bacterium, *Y. enterocolitica* has been detected in kimchi manufactured in China in 2021, making it a serious food safety issue.

Inappropriate temperature management during Kimchi cabbage production and distribution can be a major cause of bacterial growth [8]. However, studies on the predictive growth model of Kimchi, which may be contaminated with food poisoning bacteria, such as *Y. enterocolitica*, is very insufficient Therefore, in this study, we investigated the growth characteristics of *Y. enterocolitica* in Kimchi cabbage stored at different temperature and developed a predictive growth model using primary and secondary polynomial models. This model can provide basic data for hygiene management and food safety of agricultural products including cabbage.

## 2 Materials and methods

### 2.1 Bacterial strain

*Yersinia enterocolitica* (ATCC 23715) obtained from KCCM (Korean Culture Center of Microorganisms, Seoul, Korea), was used in the experiments. The bacterial stock was stored at −80 °C in tryptic soy broth (TSB; Difco Laboratories, Detroit, MI, USA) containing 30% glycerol. *Yersinia enterocolitica* (10 μL) was activated in 5 mL TSB by incubation at 37 °C for 24 h. The culture was centrifuged at 5400 rpm (4695×*g*) for 10 min at 4 °C (SUPRA22K, Hanil Science Industrial Co., Daejeon, Korea). This process was repeated twice for optimal activation of bacteria. The pellets obtained by centrifugation were resuspended in 9 mL of 0.85% sterilized NaCl solution.

### 2.2 Sample preparation and inoculation

Kimchi cabbage was purchased online, and transported on ice packs. After receipt, it was transferred to a refrigerator to 4 °C and used within 24 h. Kimchi cabbage was cut into 3 g of square pieces using sterilized scissors, and the surface was disinfected with 70% ethanol to remove the microorganisms already present in the sample.

The Kimchi cabbage samples were widely spot-inoculated to contaminate with 100 μL of the *Y. enterocolitica*. After inoculation, the samples were placed in a biological safety cabinet (CHC Lab Co. Ltd., Daejeon, Korea) to allow the *Y. enterocolitica* to stably adhere to the sample. The initial concentration of cultivated *Y. enterocolitica* was 2–2.5 log CFU/g.

### 2.3 Storage conditions and microbial enumeration

We selected ‘refrigeration storage’ as a crucial factor in related to the growth of *Y. enterocolitica* in Kimchi cabbage. The storage temperatures were set to 5 °C, 10 °C, 15 °C, and 20 °C, which are refrigerated and room temperature ranges. The samples were stored in a refrigeration chamber (SHC5000, SehanSciMed, Daejeon, Korea), and microbial analysis was performed at different time points (1–120 h). Three samples were analyzed in each experiment (repeated twice). The internal temperature of the chamber was periodically checked using thermometers both inside and outside of the device.

Kimchi cabbage samples stored at each temperature were placed in sterilized bags (Labplus Inc., Sainte-Julie, Quebec, Canada) and diluted with sterile NaCl solution for homogenization using a stomacher (Easy Mix, AES Chemunex, Rennes, France). The diluted sample (1 mL) and tryptic soy agar (TSA, Difco Laboratories, Detroit, MI, USA) were poured into petri dishes and incubated at 35 ± 1 °C for 18–24 h. Petri plates with 15–300 colonies per 1 mL of specimen solution (per 1 mL) were counted and recorded as log colony-forming units (CFU)/g.

### 2.4 Primary modeling

Baranyi model formula (Baranyi and Roberts, 1994) (Table 1) and DMFit version 3.5 (ComBase, Dresden, Germany, https://www.combase.cc/index.php/en/) were used to yield the specific growth rate (SGR, log CFU/g) values and lag time (LT, h) values of *Y. enterocolitica* of Kimchi cabbage at each storage temperature.

**Table 1:** Primary growth models used to predict the growth of *Yersinia enterocolitica* in Kimchi cabbage as a function of cold storage temperatures.

Baranyi equation
$y=y_{0}+\mu A\left(t\right)-In\;\left(1+\frac{\exp\left[\mu A\left(t\right)\right]-1}{\exp\left(y_{\max}-y_{0}\right)}\right)$
$A\left(t\right)=t+\frac{1}{\mu }In\;(\exp\left(\mu t\right)+\exp\left(-\mu \lambda \right)-\exp\left(-\mu \left(t+\lambda \right)\right)$

y = cell number (log CFU/g), y_0_ = log initial number of cells (log CFU/g), A = difference between initial and final cell numbers (CFU/g), *t* = time, *λ* = lag time, μ = maximum growth rate (log CFU/g).

### 2.5 Secondary modelings

A secondary model was calculated based on the growth of *Y. enterocolitica* at each temperature. The parameters of the primary modeling (SGR and LT values) for *Y. enterocolitica* growth data were determined by the least squares method using PROC GLM of SAS version 9.4 (SAS, Inc., Cary, NC, USA). The equations used in the secondary models were as follows [9]:

(1) y = b_0_ + b_1_T + b_2_T^2^ + ε

Where ‘y’ is the predicted value (SGR or LT), ‘b_0_’, ‘b_1_’, and ‘b_2_’ are the regression coefficients, ‘T’ is the storage temperature; and ‘ε’ is the random error.

### 2.6 Validation of the model suitability

To assess the adequacy of validation of the predictive growth model, coefficient of determination (R^2^), mean square error (MSE), bias factor (B_*f*_), and accuracy factor (A_*f*_) were used [1].

R^2^ is generally used as an overall measure for the fidelity of predictions. The equation used to calculate the R^2^ values is [10]:

R^2^ = 1 - Σei^2^/Σ(predicted values − average of the predicted values)^2^ (2).

MSE estimate the adequacy of predictive models using differences between experimental and predicted values for the specific rate of microbial growth. This value was determined using the following equation [1]:

(3) MSE = Σ Log (observed values − predicted values)^2^/n

B_*f*_ indicates that the observed values are above or below the line equivalent line compared to the average range, and if so, how large this difference. This value was determined using the following equation [11]:

B_*f*_ = 10^ΣLog(observed values/predicted values)/n^ (4).

A_*f*_ is the absolute values of the average difference between the experimentally obtained and predicted values from the secondary model. The equation used to determine the A_*f*_ is as follows [11]:

(5) A_*f*_ = 10^ΣLog |(predicted values/observed values)|/n^

Where (equation (3)−5), ‘n’ was the number of observations.

### 2.7 Statistical analysis

For all experiments, statistical significance was evaluated by repeating three times per sample. Statistical analysis was performed to determine the significant differences between the parameters calculated by primary and secondary modeling. All calculated data are presented as the average ± standard deviation. Statistical analysis was conducted using one-way analysis and Duncan's multi range test of SPSS software (version 12.0; SPSS Inc., Chicago, IL, USA), and verified at a probability level of 5% (*p* < .05).

## 3 Results and discussion

### 3.1 Growth patterns and primary modeling of Y. enterocolitica in kimchi cabbage

**Fig. 1:**
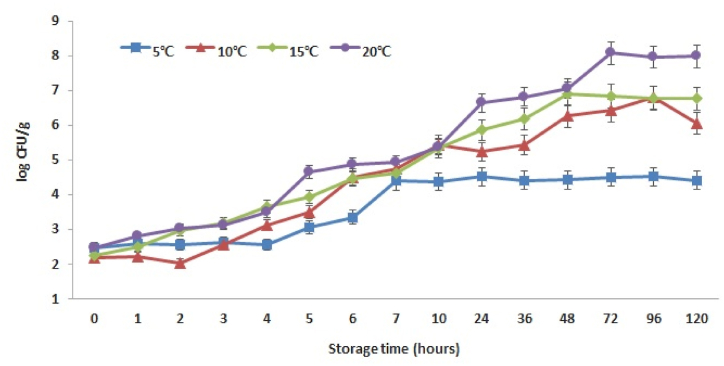
Growth curves of *Yersinia enterocolitica* in Kimchi cabbage at 5 °C, 10 °C, 15 °C, and 20 °C. Error bars indicate standard deviations of the mean of two replicates of three samples at each storage temperature and time.

In general, Kimchi cabbage is stored at a low temperature in the dark after purchase, and the quality typically does not deteriorate for 5–7 days under these conditions; therefore, we evaluated the growth pattern of *Y. enterocolitica*, a low-temperature bacterium, during this period. The growth patterns of *Y. enterocolitica* in Kimchi cabbage stored at each temperature (5 °C, 10 °C, 15 °C and 20 °C) are shown in Fig. 1. The initial concentration of *Y. enterocolitica* inoculated in Kimchi cabbage was 2.2–2.5 log CFU/g, and the number of bacteria gradually increase as the storage temperature increased. The overall growth pattern of *Y. enterocolitica* showed a typical *S*-shaped sigmoidal curve; the pattern of this bacterium stored at 5 °C showed a gentle *S*-shaped pattern. Particularly, compared to storage at other temperatures (10 °C, 15 °C, 20 °C), *Y. enterocolitica* stored at 5 °C grew slowly, and showed almost stationary growth after 7 h of storage. The number of *Y. enterocolitica* in Kimchi cabbage stored at 5 °C increased steadily by 4.4 log CFU/g during the 120-h storage. At 10 °C and 15 °C, the number of bacteria steadily increased during storage, with an increase of 4.5–4.7 log. At 20 °C, *Y. enterocolitica* had grown by more than 2 log after 5 h of storage; as the storage time increased, the number of bacteria increased rapidly, showing a value of 9.41 log CFU/g at 120 h. For *Y. enterocolitica* grown in Kimchi cabbage at different storage temperature, the maximum population density of approximately 4.5 log CFU/g was reached after 24 h of storage at 5 °C and approximately 8 log CFU/g at 72 h (3 days) at 20 °C (data not shown). Thus, the storage temperature considerably affected the maximum population density of *Y. enterocolitica* in Kimchi cabbage.

*Y. enterocolitica* infection (Yersiniosis) generally begins with the ingestion of contaminated food or water. Children under the age of 5 years are particularly vulnerable to yersiniosis, which causes gastroenteritis symptoms such as severe diarrhea and abdominal pain, and often appears as pseudoappendicitis syndrome in adults [8]. In some countries, the disease is comparable to *Salmonella* spp. As a foodborne pathogen and has raised concerns related to food safety because it can grow at low refrigeration temperatures [12]. In a study by Kim et al. (2011) [13], it was reported that 1−3 log of *Y. enterocolitica* was detected in salted cabbage, and in Korea, *Y. enterocolitica* was detected in the majority of kimchi imported from China in 2021, causing a big controversy [14]. Kowalik and Robacz (2015) [7] reported that *Y. enterocolitica* in cheese grew steadily at refrigerated temperatures of 3–9 °C. Considering these characteristics of *Y. enterocolitica*, to ensure safe storage and consumption, Kimchi cabbages should be stored at temperatures below 5 °C for a short time to effectively suppress microbial growth. The growth of *Y. enterocolitica* in Camembert cheese showed a similar pattern as our results, with approximately 4 log higher growth observed following storage at 15 °C for approximately 100 h [7]; the growth rate was slightly lower than that observed in the current study. In general, a slice of Camembert cheese has a salt content of approximately 320 mg, which may have affected the growth of *Y. enterocolitica* compared to that on fresh agricultural products such as Kimchi cabbage. *Aeromonas hydrophila* from cow milk stored at 8 °C showed rapid growth by more than 7 log after 12 h [15]. This is assumed to be because of liquid foods have suitable and stable characteristics for the activation and growth of bacteria [16]. Considering these results, bacterial growth may be affected by nutritional components (such as carbon, protein, fat) and intrinsic properties (such as pH, water activity, temperature, salt content) [3].

Based on the growth results of *Y. enterocolitica* of Kimchi cabbage stored at various temperatures (5 °C, 10 °C, 15 °C, and 20 °C), the SGR and LT values were calculated by applying the Baranyi model as the primary model. At storage temperatures of 5, 10, 15, and 20 °C, the SGR values were 0.33, 0.40, 0.60, and 0.68 log CFU/h, respectively, and the LT values were 5.63, 3.54, 2.23 and 1.09 h respectively (*p* < .05) (Table 2). As the storage temperature increased, the SGR values increased significantly (*p* < .05), and LT value decreased significantly (*p* < .05). The R^2^ values were 0.98, 0.97, 0.97 and 0.98 at 5 °C, 10 °C, 15 °C, and 20 °C, respectively, confirming that the Baranyi model was suitable for primary modeling (Table 2).

**Table 2:** Growth parameters of *Yersinia enterocolitica* in Kimchi cabbage obtained from the Baranyi model for the primary modeling.

Temperature (°C)	SGR (log CFU/h)	LT(h)	R^2^
5	0.33 ± 0.08^b^	5.63 ± 0.35^a^	0.98
10	0.40 ± 0.04^b^	3.54 ± 0.25^b^	0.97
15	0.60 ± 0.01^a^	2.23 ± 0.29^c^	0.97
20	0.68 ± 0.05^a^	1.09 ± 0.16^d^	0.98

SGR, (maximum) specific growth rate; LT, lag time; R^2^, correlation coefficient, a higher R^2^ value indicates a better fit to the data; Values are mean±standard deviations of triplicate determination. Different superscript in a column(a-d) are significant differences by Duncan's multiple range test with 5% probability.

The primary models commonly used for microbial growth curves include Baranyi model, Gompertz model [17], modified Gompertz model [18,19], logistic model [20], which have also been used in the development of commercialized modeling program. Despite considering mathematical and theoretical functions for the growth curve, these models were not originally developed for evaluating microbial growth; thus no formulas and data related to the parameters of microbial growth were included [21,22]. Therefore, Baranyi developed a mechanical model for bacterial growth that can be used with various environmental conditions and time [21,23].

Pal, Labuza and Diez-Gonzalez (2008) [24] reported that the Baranyi model best fits complex curves such as tailing phase and sigmoidal curves among several predictive models, including the modified Gompertz model and logistic linear model. Both models can be used for the sigmoid curve but the structure of the Baranyi model is looser than that of the modified Gompertz model [25]. Thus, the Baranyi model can directly yield the lag, linear, and tailing steps for several parameters, whereas the modified Gompertz equation represents the steps of the survival curves in a more complex manner [26]. Recent studies reported that the Baranyi model is used more often than the modified Gompertz model [27,28]. The Baranyi model of “combase” used in this study is a protocol based on combase's own data that predicts the survival of microorganisms (database for predictive microbiology) as a function of environmental factors such as temperature, pH, or suspension [29].

Recently, the use of GInaFiT (Geeraerd and Van Impe Inactivation Model Fitting Tool) software similar to the Baranyi model has been increasingly used to evaluate non-linear microbial growth model [30], which is being studied to be very useful in evaluating microbial survival models [31]. Therefore, in the future, research between several models that are evaluated as useful in relation to non-linear microbial evaluation is also expected to be interesting.

### 3.2 Secondary modeling and validation of Y. enterocolitica in kimchi cabbage

Secondary predictive modeling was performed to determine how the SGR and LT values derived through primary modeling can be predicted when substituted at different temperatures. Secondary modeling of the SGR and LT values was performed using the polynomial equations in SAS software as follows:

(6) SGR; y = −0.0802 + 0.0626 × T + 0.0012 × T^2^ (R^2^ = 0.96)

(7) LT; y = 7.3830 − 0.4655 × T + 0.0076 × T^2^ (R^2^ = 0.98)

(T:storage temperature).

The SGR values at 5 °C, 10 °C, 15 °C, and 20 °C yielded by the secondary polynomial equations were 0.24, 0.42, 0.59, and 0.69 log CFU/h and LT values were 5.45, 3.49, 2.12, and 1.11 h, respectively (Table 3). Furthermore, the results of secondary modeling indicated that the R^2^ values of SGR and LT were 0.96 and 0.98, respectively, which fit the model well.

**Table 3:** Growth parameters of *Yersinia enterocolitica* in Kimchi cabbage obtained from the polynomial equation for the secondary modeling.

Temperature (°C)	SGR (log CFU/h)	LT (h)
5	0.24	5.45
10	0.42	3.49
15	0.59	2.12
20	0.69	1.11

LT, lag time; SGR, (maximum) specific growth rate; NG, No Growth.

To verify the validity of the predicted value through secondary modeling, the verification results for *Y. enterocolitica* based on the temperature of Kimchi cabbage were determined, as shown in Table 4. The MSE values for SGR and LT were 0.006 and 0.000, respectively; an MSE value closer to 0 indicates a better fit of the developed predictive model [3], and thus the developed model was mathematically and statistically suitable. The B_*f*_ values for the SGR and LT were 0.919 and 0.999, respectively, suggesting that the modeling results provided a reliable prediction. The A_*f*_ values for the SGR and LT were 1.136 and 1.032, respectively, showing errors of approximately 10% but high confidence values in the other two statistical indicators; therefore, the developed model was stably calculated in terms of statistics. B_*f*_ indicates the average bias of the experimental and predicted values used to verify the results derived from the predictive model, and A_*f*_ is the average accuracy of the predicted model. In general, when the statistical indicator B_*f*_ = A_*f*_ = 1, the model is perfect [32]. In other words, In the case of the A_*f*_ indicator, a value the closer to 1 indicates a higher model fit, whereas a larger interval indicates greater inaccuracy. Additionally, if the B_*f*_ and A_*f*_ values are 0.9–1.05, the modeling is considered as “good”, 0.7–0.9 or 1.06–1.15 is “acceptable”, and <0.7 or >1.5 is evaluated as “not allowed” [33]. For example, if the calculated A_*f*_ indicator is 0.9–1.1, the model is under- or overpredicted by 10%. For MSE indicator, as mentioned earlier, a value closer to zero indicates a better fit of the developed predictive model [3]. Therefore, because both the SGR (0.006) and LT (0.000) values of MSE in this study were very close to 0 (<0.01), the model performed well.

**Table 4:** Validation of the secondary modeling using statistical indices for growth parameters of *Yersinia enterocolitica* on fresh Chinese cabbage.

		MSE	B_*f*_	A_*f*_
Validation	SGR (log CFU/h)	0.006	0.919	1.136
	LT (h)	0.000	0.999	1.032

MSE, Mean square error; A_*f*_, Accuracy factor; B_*f*_, Bias factor; LT, lag time; SGR, (maximum) specific growth rate.

Based on the results of various indicators to verify the accuracy of the developed model, the polynomial equation of the secondary modeling used in this study confirmed reliably predicted the effect of storage temperature on both SGR and LT of Kimchi cabbage. However, extrapolation of the growth predictions for LT values should be performed cautiously because the duration of LT is often considered as irregular, and evaluation of the predictive model results in a lower predictive reliability compare to the actual generation time [34] and growth data [35]. In studies by Cho et al. (2011) [36] and Park, Choi and Ha. (2019) [1], secondary modeling was performed using polynomial equation in SAS; the difference in the observed and predicted SGR values was within 0.05 log, and the difference in LT values was less than 2 min, confirming the validity of secondary modeling using the polynomial equation.

**Fig. 2:**
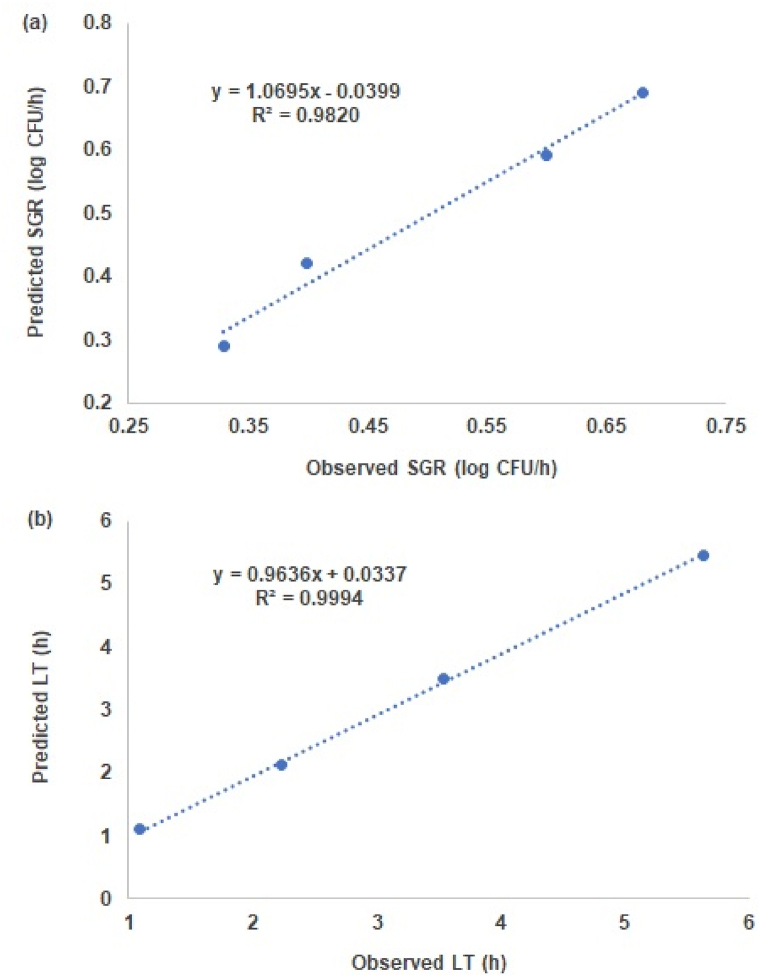
Comparative plots of the observed and predicted specific growth rate (SGR) (a) and lag time (LT) (b) for *Yersinia enterocolitica* in Kimchi cabbage in validation.

An overall comparison graph of modeling of the LT and SGR values according to the storage temperature is shown in Fig. 2. In regression analysis, the R^2^ of the SGR and LT values were 0.98 (Fig.s. 2a) and 0.99 (Fig. 2b), respectively, showing very high suitability. Scatterplots representing experimentally observed or predicted data can be used to evaluate the success of predictive model development [37]. Most points on the graph are relatively close to the 100% correlation line (y = x), indicating the excellent performance of the predictive model.

The Pathogen Modeling Program (PMP) developed by the United States Department of Agriculture was designed as a research and educational tool to estimate the effects of multiple variables on the growth, survival, and inactivation of pathogens as a predictive microbiological application. Most models used in PMP are based on experimental data on microbial behavior in broth culture. No growth model for *Y. enterocolitica* is available in PMP; therefore, modeling was applied to *Y. pseudotuberculosis*, a similar species, giving SGR values of 0.013, 0.177, 0.444, and 0.654 at 5 °C, 15 °C, and 20 °C, respectively. This differs from the results of our study because the PMP program was optimized for the *Y. pseudotuberculosis* growth in raw ground beef. In addition, despite the advances of food prediction microbiology, practical tools for these evaluations in industrial sites remain unavailable [38]. Most recent studies focusing on microbial growth responses evaluated simulated environmental conditions with optimal media conditions rather than real natural environments, including food [39]. These models may be difficult to accurately predict when applied to real foods [40,41]. Therefore, PMP is inappropriate for predict the growth of *Y. enterocolitica* in fresh agricultural products and should be further evaluated and updates.

We present a predictive growth model for *Y. enterocolitica*, which can proliferate in Kimchi cabbage during distribution, storage, and consumption as a function of refrigeration temperature. Studies of the predictive microbiology of fresh agricultural products is still insufficient; however, recent studies of growth models have extended beyond pilot experiment on broth to develop models that directly apply food-borne bacteria to real food.

The model developed in this study showed a good fit. Predicting the growth of *Y. enterocolitica* in Kimchi cabbage could be help reduce the quantitative risk of microorganisms and provide crucial information for related research and the food industry on the distribution and storage of Kimchi cabbage.

## 4 Conclusions

The predictive growth model developed using the Baranyi model in this study showed similar values as the observed results, demonstrating that it can be used as a practical model for predicting the growth of *Y. enterocolitica* based on the storage temperature of fresh Kimchi cabbage. These models can contribute to the development of an optimal model for the growth of *Y. enterocolitica* at different storage temperatures. In addition, this predictive growth model can be used to establish critical control points and critical limits in the application of hazard analysis critical control point systems to develop products using Kimchi cabbage. Furthermore, the model may be a crucial tool for controlling *Y. enterocolitica* in the production, processing, and distribution of Kimchi cabbage and can improve the safety of agricultural and agricultural products.

## Author contribution statement

Sung-Hee Park: Performed the experiments; Contributed reagents, materials, analysis tools or data; Wrote the paper. Ji Yoon Kim: Performed the experiments; Contributed reagents, materials, analysis tools or data. Eun Hae Kim, Sung Gi Min: Analyzed and interpreted the data. Shin Young Park: Conceived and designed the experiments.

## Data availability statement

Data included in article/supp. Material/referenced in article.

## Declaration of competing interest

The authors declare that they have no known competing financial interests or personal relationships that could have appeared to influence the work reported in this paper.
